# Population Structure of Non-ST6 *Listeria monocytogenes* Isolated in the Red Meat and Poultry Value Chain in South Africa

**DOI:** 10.3390/microorganisms8081152

**Published:** 2020-07-30

**Authors:** Itumeleng Matle, Thendo Mafuna, Evelyn Madoroba, Khanyisile R. Mbatha, Kudakwashe Magwedere, Rian Pierneef

**Affiliations:** 1 Bacteriology Division, Agricultural Research Council-Onderstepoort Veterinary Research, Onderstepoort 0110, South Africa; matlei@arc.agric.za; 2 Department of Agriculture and Animal Health, Science Campus, University of South Africa, Florida 1709, South Africa; mbathkr@unisa.ac.za; 3 Centre for Bioinformatics and Computational Biology, Department of Biochemistry, Genetics and Microbiology, University of Pretoria, Pretoria 0028, South Africa; MafunaT@arc.agric.za; 4 Biotechnology Platform, Agricultural Research Council-Onderstepoort Veterinary Research, Private Bag X 05, Onderstepoort 0110, Pretoria, South Africa; 5 Department of Biochemistry and Microbiology, Faculty of Science and Agriculture, University of Zululand, KwaDlangezwa 3886, South Africa; evelyn.madoroba@gmail.com; 6 Directorate of Veterinary Public Health, Department of Agriculture, Land Reform and Rural Development, Pretoria 0001, South Africa; KudakwasheM@Dalrrd.gov.za

**Keywords:** *L. monocytogenes*, subtyping, serogroups, sequence types, clone complexes, pathogenic islands, lineages, *inlA*, sequencing

## Abstract

Meat products have been implicated in many listeriosis outbreaks globally, however there is a dearth of information on the diversity of *L. monocytogenes* isolates circulating in food products in South Africa. The aim of this study was to investigate the population structure of *L. monocytogenes* isolated in the meat value chain within the South African market. Based on whole-genome sequence analysis, a total of 217 isolates were classified into two main lineage groupings namely lineages I (*n* = 97; 44.7%) and II (*n* = 120; 55.3%). The lineage groups were further differentiated into IIa (*n* = 95, 43.8%), IVb (*n* = 69, 31.8%), IIb (*n* = 28, 12.9%), and IIc (*n* = 25, 11.5%) sero-groups. The most abundant sequence types (STs) were ST204 (*n* = 32, 14.7%), ST2 (*n* = 30, 13.8%), ST1 (*n* = 25, 11.5%), ST9 (*n* = 24, 11.1%), and ST321 (*n* = 21, 9.7%). In addition, 14 clonal complex (CCs) were identified with over-representation of CC1, CC3, and CC121 in “Processed Meat-Beef”, “RTE-Poultry”, and “Raw-Lamb” meat categories, respectively. *Listeria* pathogenic islands were present in 7.4% (LIPI-1), 21.7% (LIPI-3), and 1.8% (LIPI-4) of the isolates. Mutation leading to premature stop codons was detected in *inlA* virulence genes across isolates identified as ST121 and ST321. The findings of this study demonstrated a high-level of genomic diversity among *L. monocytogenes* isolates recovered across the meat value chain control points in South Africa.

## 1. Introduction

The consumption of meat and meat-based products has increased in the last few years in South Africa (SA) [1]. This increase is primarily linked to human population growth, urbanization, higher disposable income, and a change in eating patterns as many people are adopting diets that contain high-quality animal proteins [2]. However, the chemical composition of meat predisposes it to bacterial contamination and serves as a vector for transmission of foodborne bacteria that can cause infection in humans and result in economic losses [3]. Occurrence of foodborne bacteria on meat can be due to poor animal management, slaughter practices, processing, storage conditions, and lack of meat safety knowledge [4]. Consumers need to be protected and provided with safe and wholesome products of animal origin. This can be achieved by practicing good farm animal management, proper personal hygiene, and routine surveillance of food products within the meat value chain [2]. Safe handling of meat is paramount to circumvent potential devastating effects on the health and economy of populations.

*Listeria monocytogenes* is a zoonotic foodborne bacterium that is responsible for causing a rare but potentially fatal disease known as listeriosis in humans and animals [5]. Human listeriosis has become a priority and economically important disease that contributes to public health challenges in SA and globally [6,7,8,9]. Over the years, the number of human listeriosis outbreaks and sporadic cases that emanated from various sources such as unmarked potatoes [10] and polony (Bologna sausage) [8,9] have been documented in SA. Furthermore, several studies in different geographical areas of SA have reported the presence of *L. monocytogenes* in a variety of meat products [4,11,12,13]. These studies revealed health challenges associated with *L. monocytogenes* as a result of high occurrence in food products.

The epidemic and sporadic cases of human listeriosis are commonly associated with consumption of contaminated food, particularly ready to eat (RTE) products [14]. Despite the low overall incidence of human listeriosis, this disease is linked to a high case fatality rate (20–30%) and hospitalization rates [6,15]. There is also evidence to suggest higher case fatality rates in pregnant women and individuals with neurolisteriosis [16]. The clinical manifestations of human listeriosis can range from self-limiting gastroenteritis that last a few days to more severe invasive and systematic illnesses that might be fatal in high-risk groups such as the elderly, infants, and immunocompromised people [17]. Therefore, a high percentage of the South African population is at risk as the elderly and other immunocompromised individuals contribute significantly to the total population.

The pathogenicity of *L. monocytogenes* is based on the production of the virulence factors, susceptibility of the host organism, and the virulence of a particular strain; hence, the exact infective dose and the safety margin of *L. monocytogenes* sequences is not well defined [18,19]. Several studies have provided detailed insights into the global population distribution and virulence potential of *L. monocytogenes* strains as well as the sources associated with important clonal complexes (CCs) and sequence types (STs). These have indicated an over-representation of CC1, CC2, CC4, and CC6 in clinical cases and the predominance of CC121 and CC204 in food sources [20,21,22]. In addition, the genomic characterization of the *L. monocytogenes* invasion protein (InlA) has shown a reduced virulence potential of some strains globally due to mutations associated with premature stop codons (PMSCs) [22,23]. The primary sources of *L. monocytogenes* CCs in the meat value chain are not well understood and limited data are available on the distribution of meat-associated with CCs and their virulence potential in the SA agriculture and meat value chain. 

Before the 2017–2018 outbreak of listeriosis in SA, the disease was not required to be reported and as such was not under surveillance in the country; however, a national surveillance system has since been implemented, and all isolates from human patients are analyzed by means of whole-genome sequencing (WGS) [9]. In comparison, comprehensive data on the genome characterization of *L. monocytogenes* in the food products of animal origin value chains is still lacking in SA. Matle and co-workers [13] performed an extensive national baseline survey involving nine provinces of SA to determine the occurrence of *L. monocytogenes* strains in meat and meat products in abattoirs, meat processing plants, and retail outlets. Although this study provided important information on the extent of meat contamination, the need still existed to further investigate the genomic characteristics of *L. monocytogenes* isolated from meat products using WGS in SA. The aim of this study was to subtype and characterize *L. monocytogenes* isolates recovered at selected control points in the meat value chain in SA by means of WGS.

## 2. Materials and Methods

### 2.1. Sample Information

The isolates used in this study were obtained from samples submitted between 2014 and 2019 at Agriculture Research Council-Onderstepoort Veterinary Research (ARC-OVR): Feed and Food laboratory, SA, as part of the Department of Agriculture, Land Reform, and Rural Development (DALRRD) Pathogen Profiling project number 21.1.1/VPH-01/OVI. The samples (*n* = 217) included raw meat (*n* = 55), processed meat (*n* = 126), RTE meat products (*n* = 15) and environmental samples collected from commercial pig farm environment during a listeriosis outbreak (*n* = 21). The samples originated from different animal protein sources such as beef, poultry, lamb, and pork and various food establishments (farm environments, butcheries, abattoirs, retail outlets and cold stores).

### 2.2. Isolates Categorisation

Considering the diversity of the samples from which the isolates originated, the isolates were grouped according to different categories based on the origin of the sample and the establishment of origin. The sample origin was defined as a concatenation of the type of meat product and the animal from which it was produced from. Samples collected from the farm environment were labelled as environmental samples. The number of isolates for each category is shown in Table 1.

### 2.3. Bacterial Strains and DNA Isolation

The isolates were preserved as lyophilized and were revived by inoculation into brain heart infusion (BHI) broth then incubated at 37 °C for 18–24 h. DNA was extracted from BHI broth culture using a High Pure PCR template preparation kit (Roche, Potsdam, Germany) according to manufacturer’s instructions.

### 2.4. Genome Sequencing, Quality Control and de novo Assembly

Whole-genome sequencing (WGS) of the isolates was performed at the Biotechnology Platform, Agricultural Research Council, Onderstepoort, SA. DNA libraries were prepared using TruSeq and Nextera DNA library preparation kits (Illumina, San Diego, CA, USA), followed by sequencing on HiSeq and MiSeq instruments (Illumina, San Diego, CA, USA). Quality control including adapter removal of the raw data was done using BBDuk (version 37.90; https://jgi.doe.gov/data-and-tools/bbtools/bb-tools-user-guide/bbduk-guide/). SPAdes v.3.12.0 [23] was used to create a de novo assembly of each isolate.

Multi locus sequence type (MLST) profiles were obtained from the *Listeria* database hosted by the Pasteur Institute, France (http://bigsdb.pasteur.fr/listeria/) [24]. The MLST database contains 7 loci with a total of 2069 different alleles. A k-mer based mapping tool, stringMLST [25], was used to align reads against these profiles to determine the MLST for each sequenced sample using k-mers of length 21 and 35. To validate the k-mer based predictions, all de novo assembled isolates were analyzed using MLST v.2.18.0 [26]. Serotype determination was done in silico using stringMLST and validated with blastn v.2.10.0+.

Genomes of which the in silico determined sequence type and serogroup correlated with *L. monocytogenes* were annotated using Prokka v.1.14.0 [27]. The pan-genome composition was extracted using Roary [28] and a core genome phylogenetic tree constructed with IQ-TREE v.1.6.6 [29]. Pan-genome clusters were defined as follows: Core—genes present in all isolates; soft core—genes present in at least 95% of isolates; shell-genes present between 15% and 95% of isolates; cloud-genes in less than 15% of isolates. The core genome phylogenetic tree was visualized using ggtree v1.16.6 [30].

### 2.5. Listeria Pathogenicity Islands

The presence of *Listeria* Pathogenicity Island (LIPI) in the de novo assemblies was determined for LIPI-1, LIPI-3, and LIPI-4 using blastn v.2.10.0+ with a minimum percent identity of 95% and an e-value of 1 × 10^−30^. All alleles for the abovementioned LIPI genes clusters were obtained from the *Listeria* database hosted by the Pasteur Institute, Paris, France (http://bigsdb.pasteur.fr/listeria/) [25].

### 2.6. Protein Sequence of inlA Genes

Protein sequences for the *inlA* genes were extracted from the annotated assemblies and aligned using all-versus-all blastp with an e-value of 1 × 10^−30^. The results were filtered for 99% identify and clustered using the Markov clustering algorithm (MCL) [31] with an inflation parameter of 1.8. Protein sequences were inspected for truncation based on the reference protein length of 800 amino acids (AAs).

### 2.7. Data Analysis

Analysis was done using R v.3.6.0 [32]. Proportion and association testing were done using Chi-Square tests and over-representation was indicated by a Pearson residual value of larger than 2. Diversity analysis according to ST occurrence within categories was done using the R package vegan v2.5-6 [33]. A distance matrix based on the ST count matrix was produced using vegan with the “bray” method invoked. Principle coordinate analysis was done using ape v5.3 [34] with the distance matrix as input.

## 3. Results

### 3.1. Typing Analysis

**Figure 1:**
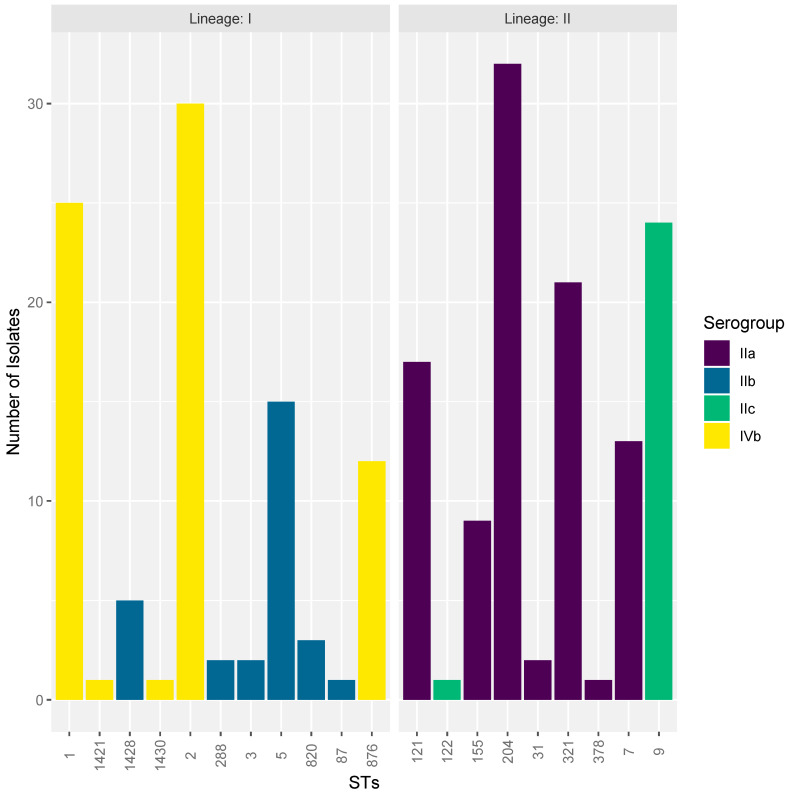
Lineage, serogroup, and sequence type distribution of *Listeria monocytogenes* isolates.

The isolates were grouped into different STs, 20 in total, and classified as either Lineage I or II (Figure 1). Eleven lineage I and nine lineage II STs were identified with lineage I accounting for 44.7% (*n* = 97) and lineage II accounting for 55.3% (*n* = 120) of the isolates. Five STs (25% of all STs) were found to be singularly represented in the isolates. The five most frequent STs were ST204 (*n* = 32, 14.7%), ST2 (*n* = 30, 13.8%), ST1 (*n* = 25, 11.5%), ST9 (*n* = 24, 11.1%), and ST321 (*n* = 21, 9.7%), respectively. The other identified STs are presented in Table 1. Fourteen CCs were identified of which six were in lineage I and eight in lineage II, with CC1 (*n* = 38, 17.5%) the most prevalent followed by CC204 (*n* = 32, 14.7%) and CC2 (*n* = 31, 14.3%). Four serogroups were identified in the 217 isolates, with serogroup IIa (*n* = 95, 43.8%) being the most prevalent, followed by IVb (*n* = 69, 31.8%), IIb (*n* = 28, 12.9%), and IIc (*n* = 25, 11.5%) (Figure 1).

**Figure 2:**
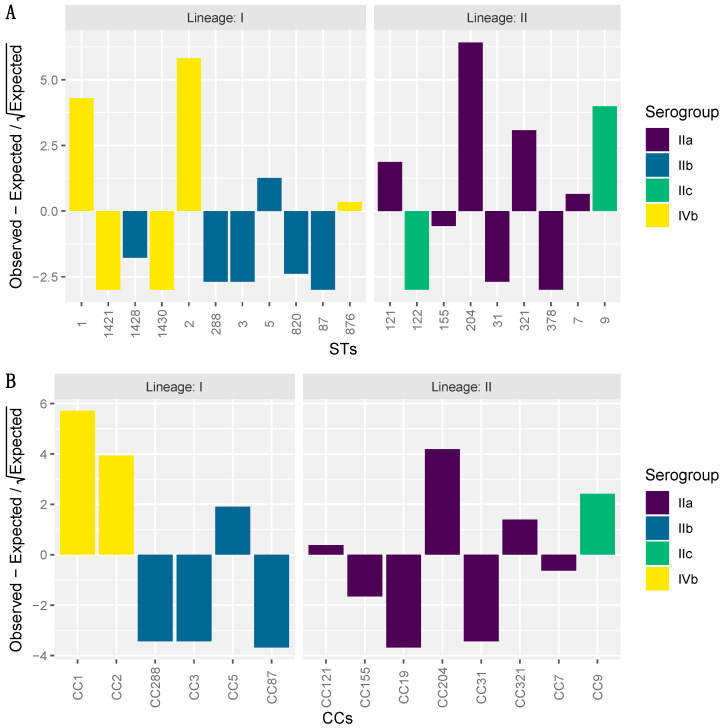
Hanging chi-gram indicating the deviation from expected occurrence across the *Listeria monocytogenes* isolates: (**A**) Sequence type; (**B**) clonal complex.

The distribution of lineages, serogroups, CCs, and STs among the 217 isolates were tested using a Chi-Square goodness of fit test. The test results indicated that the serogroups (*p*-value = 1.283 × 10^−13^), CCs (*p*-value = 5.761 × 10^−24^) and STs (*p*-value = 3.327 × 10^−32^) were not commonly distributed among the samples (Figure 2). In particular, serogroups IIa (lineage II) and IVb (lineage I) were found to be over-represented. The STs that exceeded the expected distribution were ST1, ST2, ST9, ST204, and ST321, which all belonged to serogroups IIa and IVb with the exception of ST9 in serogroup IIc.

### 3.2. Samples Categories Analysis

Sample origin contained 12 categories (Table 1) and a Chi-Square test of independence were used to identify significant associations between the categories and the isolate typing results. Serogroup IIb in the “RTE-Poultry”, IVb in the “Processed Meat-Beef”, and IIc in the “Environmental Sample”, “Abattoir” and “Farm” groups were found to be over-represented (*p*-value = 0.003). For the STs, over-representation was detected for “Processed Meat-Pork” (ST876), “Raw-Beef” (ST378), “Raw-Lamb” (ST121), “Raw-Pork” (ST122), “Raw-Poultry” (ST5), “RTE-Pork” (ST121), “RTE-Poultry” (ST3), and “Environmental Samples” (ST7, ST9, ST31, ST155, and ST288) (*p*-value = 1.771 × 10^−11^). In the Establishment category, serogroup IIc was found to be significantly over-represented in Abattoirs and Farms (*p*-value = 0.009). Abattoirs were further found to be significantly associated with ST122; “Butcheries” with ST820; “Cold Stores” with ST121; Farms with ST7, ST9, ST31, ST155, and ST288; and “Processing Plants” with ST2 and ST3 (*p*-value = 8.602 × 10^−13^).

**Figure 3:**
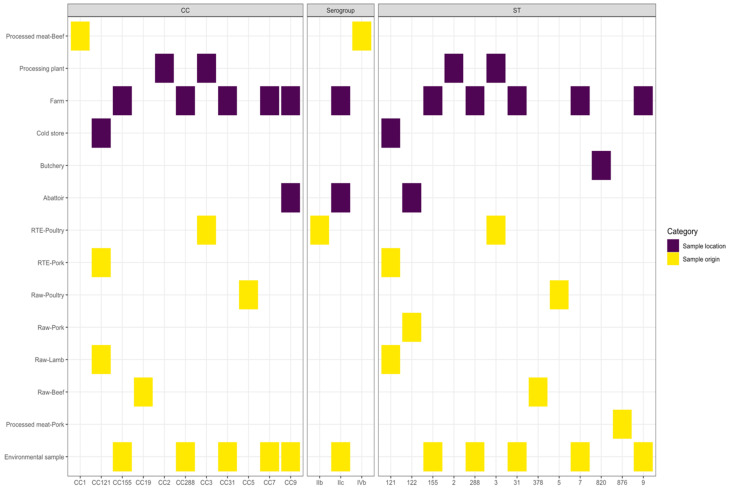
Over-representation of serogroups, clonal complexes (CCs) and sequence types (STs) across all categories.

Analysis of CCs and over-representation in the various categories indicated 11 CCs, which were deemed to be associated with a certain category. In the sample origin category, the “Environmental samples” group displayed over-representation of various CCs, which were CC7, CC9, CC31, CC155, and CC288 (*p*-value = 2.222 × 10^−16^). These CCs were further found to be over-represented in Farms (*p*-value = 2.658 × 10^−8^). The “Processed Meat-Beef” category had an over-representation of CC1; CC9 in Abattoirs; and in the “Processing Plant” establishment, CC2 was more than what was expected. In the “RTE-Poultry” and “Processing Plant” categories CC3 was over-represented, CC5 in the “Raw-Poultry” and C19 in “Raw-Beef” categories with CC121 significantly abundant in “Raw-Lamb”, “RTE-Pork”, and “Import Cold Stores”. Over-representation of serogroups, STs, and CCs, indicated by a Pearson residual value larger than 2, for all categories, is presented in Figure 3.

### 3.3. ST Diversity Analysis

**Figure 4:**
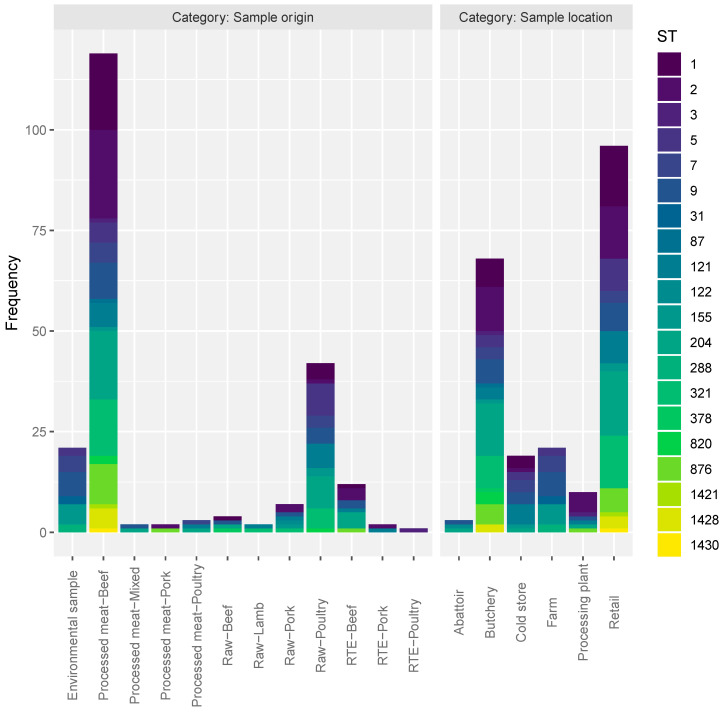
Frequency of STs per sample collection category.

**Figure 5:**
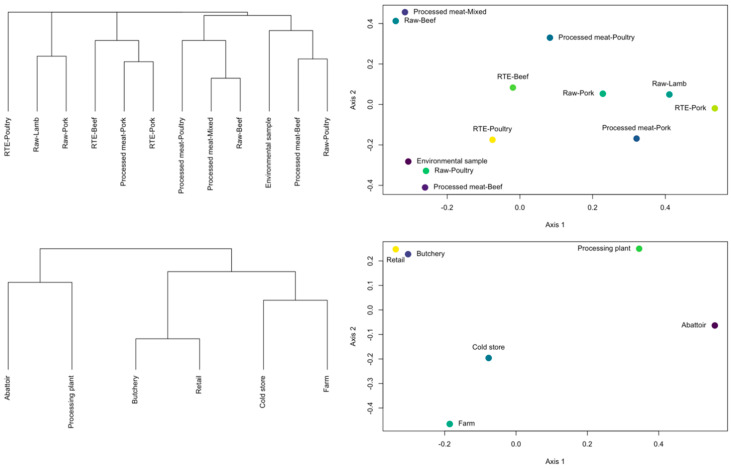
Hierarchical clustering dendograms and Principle coordinates analysis for the different sample categories.

The ST results were transformed into a count matrix and diversity analysis done according to the sample collection categories. The frequency of STs per category is displayed in Figure 4. The values for four different diversity indices (Richness, Simpson, Shannon, and Inverse Simpson) are presented in Table 2 for both the categories “Sample origin” and “Sample location”. Samples from the “Processed meat-Beef” category displayed the highest ST diversity, as indicated by all the indices, with “Raw-Poultry” having the second highest diversity. With regards to the “Sample location” it was found that the “Butchery” category had the highest ST diversity, closely followed by the “Retail” category. Results of clustering analysis and Principle coordinates analysis (PCOA) of the sample categories and the ST occurrence are displayed in Figure 5. In general, the “Sample origin” categories “Processed Meat-Beef”, “Raw-Poultry”, and “Environmental sample” formed a cluster with “Processed meat-Mixed” and “Raw-Beef” grouping together. In the “Sample location” category, “Butchery” and “Retail” grouped closely together.

### 3.4. Pathogenicity Islands

The *actA*, *hly*, and *mpl* genes, which form part of the LIPI-1 gene cluster were present in all the sequenced isolates. The complete gene cluster of LIPI-1 was present in 16 (7.4%) isolates all of which were found exclusively in “Raw-Poultry” and “Processed meat-Beef” categories obtained from “Butchery”, “Cold Stores”, and “Retail” Sample locations (Supplementary Table S1). These isolates presented CCs from lineage I (CC1, CC2 and CC5) and lineage II (CC9, CC121, CC155, and CC321). The complete LIPI-3 gene cluster was identified in 47 (21.7%) isolates (lineage I: 95.7%; lineage II: 4.3%), of which 35 (74.5%) originated from the “Processed meat-Beef” category. Four CCs (CC1, CC2, CC3, and CC288) belonged to lineage I and lineage II were represented only by CC204. A complete LIPI-4 gene cluster was detected in four isolates (1.8%) with the majority (75%) found in serogroup IVb (CC2 and CC87) and all in lineage I.

### 3.5. Protein Sequence of inlA

**Figure 6:**
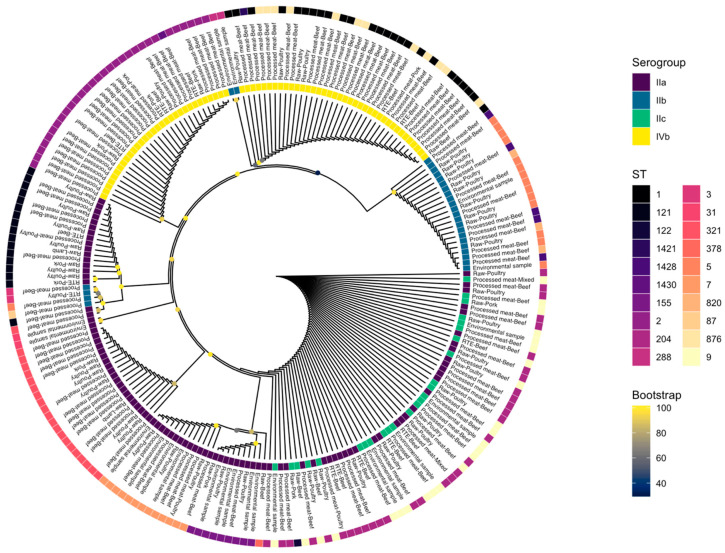
Phylogenetic analysis of *inlA* gene sequences obtained from isolates in this study.

Eight different *inlA* groups (1–8) were identified from the 217 sequenced isolates in this study (Figure 6 and Supplementary Table S2). Group 1 (size = 100) and group 2 (size = 93), harbored diverse STs, which all belonged to lineage II and lineage I, respectively. A total of 18 InlA protein sequences were found to be truncated with lengths range from 491–699 AAs. All the proteins in cluster 3 (size = 14, ST121) and cluster 6 (size = 2, ST121) were found to be truncated as well as the proteins of the singleton clusters 7 (size = 1, ST121) and 8 (size= 1, ST321). All truncated proteins belonged to isolates from lineage II, serogroup IIa, which were obtained across different establishments (“Butchery”, “Retail”, “Processing Plant”, and “Cold Store”) and sample origin (“Raw-Pork”, “Raw-Lamb”, “Raw-Poultry”, “Processed Meat-Beef”, “Processed Meat-Poultry”, and “RTE-Beef”) categories.

### 3.6. Core Genome Phylogeny

**Figure 7:**
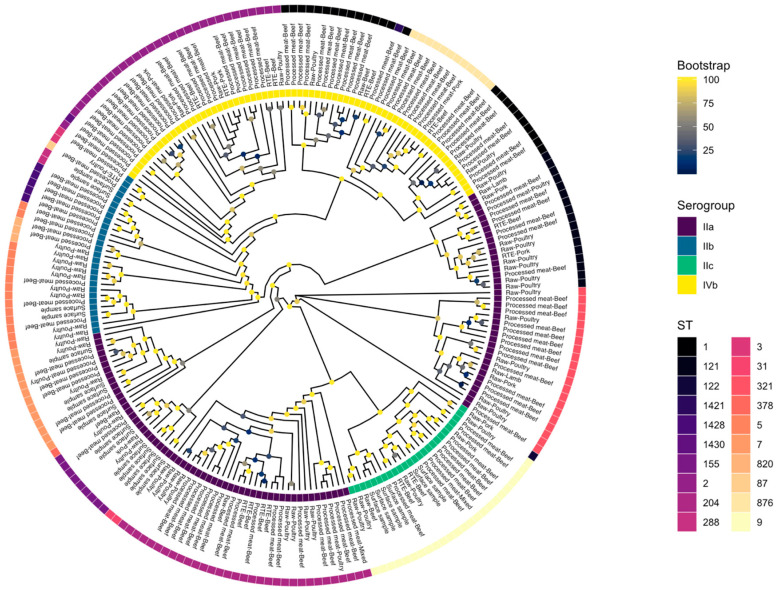
Phylogenetic analysis of *Listeria monocytogenes* isolates based on the core genome.

In total, 22,790 genes were predicted across the 217 *L. monocytogenes* isolates. The partitioning of genes across the pan-genome was as follows: core – 1029 genes; soft core – 1141 genes; shell – 1711 genes; cloud – 18,909 genes. Phylogenetic analysis, based on the core genome, is displayed in Figure 7.

## 4. Discussion

To have a better understanding of population structure and genomic diversity of *L. monocytogenes* isolates in SA, a total of 217 isolates representing different meat and meat products as well as environmental samples were characterized using WGS. WGS is a very powerful tool for the characterization of *L. monocytogenes* as it allows an unprecedented subtyping resolution by using the entire genome to determine strain diversity and virulence traits [35,36,37]. The findings of the present study give a detailed overview into the genomic diversity of *L. monocytogenes* in the meat value chain that can inform food safety risk-based decisions and risk assessment.

The primary and universally acceptable method for characterization of *L. monocytogenes* isolates has been serotyping [38]. Serotyping has been used as a rapid tool for epidemiological investigations of listeriosis outbreaks and to understand the importance of certain serotypes in causing listeriosis in humans [39]. Analysis of the serotypes in this study revealed that all isolates belonged to four major serogroups IIa, IVb, IIb, and IIc (43.8%, 31.8%, 12.9%, and 11.5%, respectively). This is in general agreement with observations made in other countries where serogroup IIa, IIb, and IVb isolates were found frequently while serogroup IIc isolates were rarely found [39,40,41]. In Ireland, O’Connor et al. [42] analyzed 5869 of *L. monocytogenes* isolates from different foods and found that the most common serogroup was IIa (43.9%), followed by IVb (27.5%), IIb (16.1%), and IIc (12.2%).

In a comparative analysis of serogroups, a hierarchy among isolates was observed with IIa and IVb found to be over-represented. The high presence of serogroup IIa in this study was expected, as IIa has been previously identified as over-represented in food sources and environmental samples in different countries [22,41,43]. Although serogroup IIa is highly associated with contamination of food, it is important to mention that they can cause human infection in certain countries with a high number of susceptible individuals such as SA [44]. Over-representation of serogroup IVb in the present study is concerning as more than 80% of human infections globally are caused by *L. monocytogenes* strains in this serogroup [22,45,46]. Further analysis of serogroup distribution based on sample origin indicated over-representation (*p*-value = 0.003) of IIb in “RTE-poultry”, IVb in “Processed meat”, and IIc in “Environmental samples”. This distribution provides critical information on the meat products that are prone to contamination by certain serogroups of *L. monocytogenes* and may subsequently help in good agricultural and hygiene practices, policy formation, and control measures of this bacterium in South Africa. For instance, implementing proper biosecurity and biosafety measures as good agriculture practice at farm level can play a critical role in minimizing the introduction and spread of different serogroups of *L. monocytogenes* on downstream processing steps across meat value chain.

MLST is a technique used to analyze nucleotide sequence data from a number of conserved (usually 7) housekeeping genes to derive a combination of alleles known as a ST. The application of this technique in the present study has served as tool also to determine the lineages and CCs of *L. monocytogenes*. CCs are defined as a group of STs differing by no more than one allele from at least one other ST in the group, regardless of its involvement in outbreaks [22]. Analysis of MLST data revealed the distribution of 20 different STs among all the sample isolates that belong to two main lineages, I or II. Similar descriptive differentiation of lineage I and lineage II isolates has been recorded in previous studies [22,47]. This suggests that lineage I and II isolates are important etiological agents common in the South African red meat and poultry value chain. The five largest ST groupings identified in this study, ST204 and ST321 (serogroup IIa), ST1 and ST2 (serogroup IVb), and ST9 (serogroup IIa), have previously been isolated from meat, meat products and production environments around the world [40,47,48,49]. However, this is the first detailed report on the distribution of STs along the livestock value chain in SA and as such it provides contemporary and applicable data. The predominant ST in the current study, ST204, has also been reported by Kwong et al. [50] and Ebner et al. [51], as the most common ST in meat-associated products in Australia and France. Other studies reported ST204 as a common persisting strain of *L. monocytogenes* that has been isolated from various sources such as food processing facilities [52], non-clinical isolates [22], and RTE food products [53]. ST1 and ST2 are regarded as the most common STs associated with food contamination and causing infection of humans and animal globally [54,55,56]. In a survey of food-producing facilities between 1996 and 2003 in Austria, ST1 and ST2 were the most predominant in meat-based products as cited by Ebner et al., [51]. Data on the occurrence and distribution of ST9 and ST321 in meat and meat products are lacking globally.

In comparison to the STs in the meat value chain, the non-ST6 sequence types reported from molecular epidemiology of human cases in South Africa are ST1, ST2, ST5, ST54, ST204, ST876, ST7, ST219, Unknown ST, Novel ST, ST101, ST1039, ST224, ST3, ST554, ST8, ST808, ST88, and ST87 in order of frequency [57]. ST6, ST132, ST155, ST2, ST204, ST3, ST5, ST533, ST602, and ST9 have been reported as the common environmental STs in SA can food production facilities [9,57]. Some of these STs (ST2, ST3, ST5, ST9, ST155, and ST204) have been reported in the present study and are induced with mechanisms that allow them to survive in food production environment and keep contaminating food products [5]. Therefore, there is need to link clinical isolates to food samples, since such epidemiological linkages are known to help further understand the key transmission routes and high-risk foods [22].

The absence of highly hypervirulent strains of *L. monocytogenes* ST6 was observed in the present study. In SA, ST6 strains have been associated with RTE products as samples cultured from a meat production facility’s food contact and non-contact environmental surfaces yielded ST6 isolates, which, together with the isolates from the human patients, belonged to the same core-genome MLST cluster with no more than four allelic differences [9]. The absence of ST6 in the current study and the rapid decline in the incidence of *L. monocytogenes* ST6 infections in humans soon after a recall of the implicated RTE processed meat products suggests that polony (Bologna sausage) produced at a single facility was highly likely to be the outbreak source with the primary contamination originating from a confined primary source [9].

Diversity analysis performed according to the sample collection categories (“Sample origin” and “Sample location”) showed that isolates from “Processed Meat-Beef” and “Butchery” categories harboured more heterogonous STs (ST1, ST2, ST3, ST5, ST7, ST9, ST87, ST121, ST155, ST204, ST321, ST820, ST876, and ST1428) of *L. monocytogenes*. It was also observed that isolates from “Raw-Poultry” and “Retail” categories harbored the second highest diversity of STs (ST1, ST5, ST9, ST155, ST204, and ST321). The clustering observed between “Processed Meat-Beef/Raw-Poultry” and “Butchery/Retail” categories based on the ST occurrence is highly comparative with several previous studies that recorded more diversity in isolates from RTE products [20,58]. Although, human infections caused by *L. monocytogenes* are commonly linked to RTE products, the findings of the present study are important in the South African context as raw-meat and processed-meat products are part of the raw materials for RTE.

The isolates in the present study were also classified into 14 CCs that represent two typical groups of *L. monocytogenes* CCs. The first group (infection-associated isolates) includes isolates (CC1, CC2, CC4, and CC6) that belong to lineage I and have a strong link to clinical cases (also known as hypervirulent strains) while the second group (food-associated isolates) represent isolates (CC7, CC9, CC121, CC155, and CC204), which belong to lineage II and are predominantly found in the food production environment [58]. The distribution of infection-associated isolates revealed the presence of CC1 and CC2, which were found to be over-represented in the “Raw-Beef” and “Processing Plant” categories, respectively. This over-representation of CC1 and CC2 clones in meat samples has been reported in different studies globally, which suggest their adaptation to diverse food products [25,59]. The distribution of food-associated isolates of *L. monocytogenes* CCs revealed a significant over-representation of CC7, CC155, CC9, CC121, and CC204, which all belong to lineage II. CC7 and CC155 were mostly found in isolates recovered from farm and environmental samples. CC7 isolates have been globally reported from diverse sources such as animal (wild, poultry, ruminants and fish), abattoir floor, compost, animal food products (milk, cheese, meat), and animal feeds (hay, silage) suggesting the possibility that it might persist in varying environments [39,60,61,62]. CC155 isolates were frequently detected in food samples in Eastern Asia [63], animals in Switzerland [64], and to a lesser extent in clinical cases in France, New Zealand, Greece, and Netherlands [65]. In the present study, CC9 and CC121 were significantly abundant in samples from “Raw-Lamb” and “Cold Stores (raw imported meat samples) categories and over-represented in “RTE-Pork” meat. Other studies also reported CC9 and CC121 as being significantly over-represented in food of animal origin and food-processing facilities around the world [47,52,66,67]; however, in this study, no significant association of CC204 isolates were observed with respect to environmental samples as well as meat and meat products isolates.

In the current study, although there was a bias towards specific lineages and CCs, there was considerable variation on pathogenic islands known to contribute to *L. monocytogenes* virulence among the isolates. The complete LIPI-1 gene cluster was detected in 16 isolates, recovered exclusively from “Raw-Poultry” and “Processed meat-Beef” categories. The presence of LIPI-1 in these meat products indicate an increased potential risk to cause infection in humans as LIPI-1 harbor a cluster of genes, *prfA*, *plcA plcB*, *hly*, *mpl*, and *actA*, that are very important in the infectious cycle of *L. monocytogenes* [68]. LIPI-3 consists of eight genes (llsAGHXBYDP) and this complete gene set was detected in 47 isolates, of which the majority (95.7%) were from lineage I (CC1, CC2, CC3, and CC228) and originating from the “Processed meat-Beef” category. This island encodes for hemolysin listeriolysin S, which is known to contribute to the survival of *L. monocytogenes* in human polymorphonuclear neutrophils [69]. Therefore, the presence of LIPI-1 and LIPI-3 islands in isolates from the “Processed meat-Beef” category, increases the risk for certain members of the population, such as the elderly, acquiring listeriosis in SA. LIPI-4 island is often implicated in placental and central nervouss system infections [69,70] and was found in four isolates in the present study. LIPI-4 has previously been identified mostly in CC4 isolates, however results of this study indicate its presence in CC2 and CC87 isolates, which is consistent with a recent report of this island in CC2 and CC87 isolates cultured in China [70]. The presence of LIPI-4 islands in hypervirulent CC2 and CC87 *L. monocytogenes* strains in beef and pork products must be a consideration in public health risk management.

The *inlA* gene encodes a surface protein that is responsible to facilitate the invasion of human intestinal epithelial cells by *L. monocytogenes* [71]. However, truncation of the *inlA* gene due to premature stop codons (PMSCs) has been associated with reduced invasiveness in some *L. monocytogenes* STs that possess them [71]. Analysis of the inlA protein sequence from isolates in this study identified 18 isolates, all from ST121 (*n* = 17) and ST321 (*n* = 1), having PMSCs, both of which were lineage II STs. Although the ST121 and ST321 PMSC mutation has previously been reported [22,64], this is the first report in SA.

## 5. Conclusions

Characterization of *L. monocytogenes* isolates from 2014–2019 using WGS has provided valuable insights into strain diversity and virulence potent of isolates found in meat products consumed in SA. This is also the largest study to report baseline data on the presence of *L. monocytogenes* serogroups, lineages, STs, and CCs across meat value chain in SA. This study confirmed the heterogeneous distribution of *L*. *monocytogenes* CCs across different meat and meat products with evidence of over-representation of certain CCs, which share similarities with those previously linked with human listeriosis outbreaks in other geographical areas. This study again illustrated meat products which are prone to contamination by diverse strains of *L. monocytogenes* within a specific point in the value chain. This study highlights the association of multiple STs of *L. monocytogenes* to different meat products in SA and identifies virulence traits as well as genetic mutations of certain subgroups found in food products. Therefore, the information generated here can be used in food safety risk assessment, management and protecting public health.

Future work is still required to compare the WGS dataset produced from this study with clinical isolates from the same timeframe and geographic regions, to identify clusters and determine potential linkages to human listeriosis cases and outbreaks, taking into consideration temporal, microbiological, and epidemiological evidence.

## Figures and Tables

**Table 1:** Number of isolates for each category together with the different STs and serogroups found in each category.

Establishment	Sample Origin	Number of Isolates	STs	CCs	Serogroups
Farm (*n* = 21)	Piggery Environment samples	21	ST5, ST7, ST9, ST31, ST155, ST288	CC5, CC7, CC9, CC31, CC155, CC288	IIa, IIb, IIc
Abattoir (*n* = 3)	Processed meat-Beef	1	ST9	CC9	IIc
	Raw-Pork	1	ST122	CC9	IIc
	Raw-Poultry	1	ST204	CC204	IIa
Butchery (*n* = 68)	Processed meat-Beef	53	ST1, ST2, ST3, ST5, ST7, ST9, ST87, ST121, ST155, ST204, ST321, ST820, ST876, ST1428	CC1, CC2, CC3, CC5, CC7, CC9, CC87, CC121, CC155, CC204, CC321	IIa, IIb, IIc, IVb
	Processed meat-Mixed	1	ST9	CC9	IIc
	Processed meat-Poultry	3	ST7, ST121, ST204	CC7, CC121, CC204	IIa
	Raw-Beef	1	ST378	CC19	IIa
	Raw-Pork	1	ST121	CC121	IIa
	Raw-Poultry	3	ST5, ST204, ST820	CC5, CC204	IIa, IIb
	^1^ RTE-Beef	6	ST2, ST9, ST204	CC2, CC9, CC204	IIa, IIc, IVb
Cold store (*n* = 19)	Raw-Beef	1	ST9	CC9	IIc
	Raw-Poultry	18	ST1, ST2, ST5, ST7, ST9, ST121, ST155, ST204	CC1, CC2, CC5, CC7, CC9, CC121, CC155, CC204	IIa, IIb, IIc, IVb
Processing plant (*n* = 10)	Processed meat-Beef	2	ST2, ST9	CC2, CC9	IIc, IVb
	Processed meat-Pork	2	ST2, ST876	CC1, CC2	IVb
	Raw-Pork	2	ST2	CC2	IVb
	^1^ RTE-Beef	1	ST204	CC204	IIa
	RTE-Pork	2	ST2, ST121	CC2, CC121	IIa, IVb
	^1^ RTE-Poultry	1	ST3	CC3	IIb
Retail (*n* = 96)	Processed meat-Beef	63	ST1, ST2, ST5, ST7, ST9, ST121, ST204, ST321, ST876, ST1421, ST1428, ST1430	CC1, CC2, CC5, CC7, CC9, CC121, CC204, CC321	IIa, IIb, IIc, IVb
	Processed meat-Mixed	1	ST204	CC204	IIa
	Raw-Beef	2	ST1, ST204	CC1, CC204	IIa, IVb
	Raw-Lamb	2	ST121, ST321	CC121, CC321	IIa
	Raw-Pork	3	ST9, ST155, ST321	CC9, CC155, CC321	IIa, IIc
	Raw-Poultry	20	ST1, ST5, ST9, ST121, ST155, ST204, ST321	CC1, CC5, CC9, CC121, CC155, CC204, CC321	IIa, IIb, IIc, IVb
	^1^ RTE-Beef	5	ST1, ST2, ST121, ST204, ST876	CC1, CC2, CC121, CC204	IIa, IVb

^1^ RTE—Ready to Eat.

**Table 2:** Diversity indices based on the occurrence of STs in the different categories.

Category	Samples	Richness	Shannon	Simpson	Inverse Simpson
Sample origin	Processed meat-Beef	16	2.357795795	0.884824518	8.682403433
	Processed meat-Mixed	2	0.693147181	0.5	2
	Processed meat-Pork	2	0.693147181	0.5	2
	Processed meat-Poultry	3	1.098612289	0.666666667	3
	Raw-Beef	4	1.386294361	0.75	4
	Raw-Lamb	2	0.693147181	0.5	2
	Raw-Pork	6	1.747868097	0.816326531	5.444444444
	Raw-Poultry	10	2.122400638	0.866213152	7.474576271
	^1^ RTE-Beef	6	1.632630927	0.777777778	4.5
	RTE-Pork	2	0.693147181	0.5	2
	RTE-Poultry	1	0	0	1
	Environmental sample	6	1.687293537	0.798185941	4.95505618
Sample location	Abattoir	3	1.098612289	0.666666667	3
	Butchery	15	2.405603569	0.890138408	9.102362205
	Cold store	8	1.927544531	0.836565097	6.118644068
	Farm	6	1.687293537	0.798185941	4.955056179
	Processing plant	6	1.497866137	0.7	3.333333333
	Retail	13	2.30089177	0.885416667	8.727272727

^1^ RTE—Ready to Eat.

## Supplementary Materials

The following are available online at https://www.mdpi.com/2076-2607/8/8/1152/s1, Table S1: *Listeria* pathogenic islands metadata, Table S2: InlA protein sequence clusters metadata.
